# Forward Voltage Drop Induced by an Abnormal Threading Dislocation Aggregation in 4H-SiC GTO Devices

**DOI:** 10.3390/ma12244042

**Published:** 2019-12-05

**Authors:** Yingxin Cui, Peng Dong, Zhe Chen, Zhiqiang Li, Lianghui Li, Juntao Li

**Affiliations:** 1 Microsystem and Terahertz Research Center, China Academy of Engineering Physics, Chengdu 610200, China; cuiyingxin@mtrc.ac.cn (Y.C.);; 2 Insititute of Electronic Engineering, China Academy of Engineering Physics, Mianyang 621999, China

**Keywords:** 4H-SiC, gate turn-off thyristor, threading dislocation aggregation, forward voltage drop

## Abstract

An abnormal star-like defect was found on the failed SiC gate turn-off thyristor (GTO) devices after metal removal and KOH etching at 450 °C in this work. It is of extraordinary larger size of 210–580 µm, even much larger than the etch pit of a micropipe in 4H-SiC. In addition, the abnormal star-like defect, exhibiting the consistent orientation with the six-fold symmetry of silicon carbide, was found to consist of several penetrating dislocations with the help of a LEXT OLS4000 3D laser confocal microscope. These abnormal star-like etch pits can severely reduce the forward blocking characteristic of GTOs, while exerting insignificant influence on the forward current-voltage characteristics between anode and gate electrode of the 4H-SiC GTO devices. Interestingly, the relationship between forward voltage drop and dislocation density is affected by the abnormal star-like defect. A regular increase of forward voltage drop at 100 A/cm^2^ was observed with the increasing dislocation density, while this correlation disappears in the presence of an abnormal star-like defect.

## 1. Introduction

Silicon carbide (SiC) is an attractive material for semiconductor devices operating under extreme conditions. This is because of its extraordinary physical properties, such as high electrical break down field, high thermal conductivity, and high electron saturation velocity [1,2,3]. Among the many kinds of SiC devices, specific interest has been focused on the 4H-SiC gate turn-off thyristor (4H-SiC GTO) for pulsed-power applications because of its advantages in high voltage, high-current, and high temperature applications over silicon-based equivalent devices [4,5]. The conductivity modulated drift region of the GTO allows high voltages and high current densities while maintaining a low forward voltage [6]. This conductivity modulation effect reduces the resistance of the thick, lowly-doped drift region that is required for blocking high voltages. This is achieved by the injection of carriers from adjacent device regions. However, the degree of conductivity modulation is heavily dependent on the carrier recombination from the point defects and extended defects in the SiC material, as such increasing the resistance of the drift region and hence the overall on-state power losses of the device. Therefore, it is of key importance to have a good knowledge of defects generation in the SiC material, which plays a crucial role in the well-known bipolar degradation in bipolar devices.

Although continuous improvement has been made in SiC epitaxial growth, such as the high uniformity in doping concentration and remarkable reduction of epi-defects (trianglar 3C inclusion and carrot defect) [7,8], the density of extended defects in SiC epi-wafer is still on the order of 10^2^–10^4^ cm^−2^. The size and performance of SiC devices, especially for high-voltage bipolar devices, are still severely hindered by the extended defects, surface defects, and interfacial dislocations of SiC substrate and its epitaxial layers. In addition, the generation and propagation of defects in the process of device preparation, such as stacking faults, basal plane dislocations and point defects, are considered to degrade the electrical characteristics of power devices and their reliability. The influence of surface pit shape on 4H-SiC double implanted metal-oxide-semiconductor field-effect transistor (MOSFETs) reliability under a high temperature drain bias test has been investigated by Uchida. et al. [9] in 2015, and the failure was revealed to be an oxide breakdown above the pit generated at the threading mixed dislocation in both pit shapes. Bonyadi. et al published the impact of a trianglar 3C inclusion on fabricated 4H-SiC PiN diodes, and they found that the existence of 3C triangular inclusions limit the active area of the devices and create a short path through the drift region which increases the leakage current almost 10^8^ times higher than the devices fabricated on epitaxial layer with low defect density [10].

In this work, an abnormal star-like defect was found after metal removal and KOH etching at 450 °C on the failed SiC GTO devices, which consists of several penetrating dislocations and exhibits the consistent orientation with the six-fold symmetry of silicon carbide. Its formation mechanisms and influence on the forward and blocking characteristic of SiC GTOs are investigated.

## 2. Materials and Methods 

**Figure 1:**
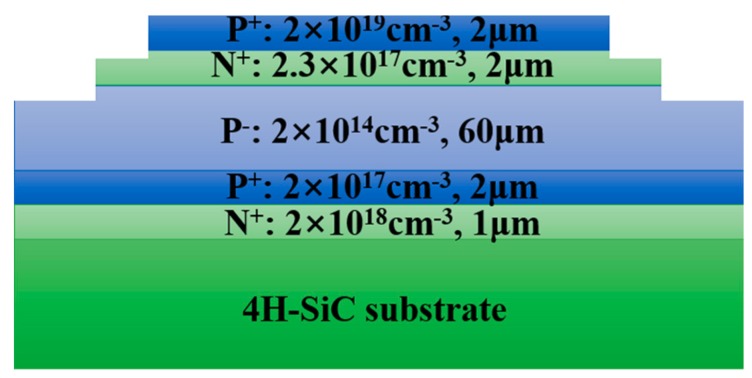
Schematic cross-section view of a 1.0 cm^2^ SiC gate turn-off thyristor (GTO). The GTO has a 60 µm drift layer thickness.

Figure 1 shows the schematic cross-section view of a 1.0 cm^2^ SiC GTO. The conventional asymmetric blocking p^+^np^−^pn structure is used for the 4H-SiC GTO device. The five epitaxial layers were grown on an 4°-off n-type 4H-SiC wafer from Ascatron AB, Inc., Kista, Sweden. A 60 µm thick drift layer was then grown on the substrate, with a doping concentration of about 2 × 10^14^ cm^−3^. The highly doped anode layer (p^+^∼2 × 10^19^ cm^−3^) is 2 µm and the gate layer (n^+^∼2.3 × 10^17^ cm^−3^) is 2 µm thick. The top p^+^ epilayer was first etched down to the base region. Nitrogen atoms were then implanted to form gate contact, followed by the creation of an n-type single zone junction termination extension (JTE) region by nitrogen implantation and activation anneal. Finally, the ohmic contacts to the anode, gate, and the substrate of the devices were formed by rapid thermal anneal and metal deposition. Figure 1 shows the schematic cross-section view of a 1.0 cm^2^ SiC GTO. The GTO has a 60 µm drift layer thickness.

The forward blocking and on-state characteristics for 1.0 cm^2^ GTOs were measured by a Keysight B1505 Semiconductor Parameter Analyzer (Keysight, CA, America) at room temperature. The negative voltage was applied to the cathode, while the anode is grounded. The applied trigger current on the gate was −100 mA. For forward blocking characterization, the applied voltages on the cathode was ranging from −3 KV to 0 V, which in this case was limited by the capability of our present B1505 Semiconductor Parameter Analyzer.

As some of the SiC GTOs exhibit lower breakdown voltage, the destructive physical analysis is then performed. Before the preferential etching of defects in the anode and gate layer, the Ni, Ni/Ti/Al alloy, and silicon dioxide passivation layer on the surface of anode and gate layer need to be eliminated. Thus, the GTO samples were repeatedly immersed in analytical reagent (AR) grade hydrochloric acid and 10% hydrofluoric acid solution, followed by ultrasonic cleaning in acetone, ethanol, and deionized water, each for 5−10 min, until the silicon carbide surface was exposed. Then, the preferential etching was carried out in the following process. A Ni-crucible filled with KOH granules of >85% purity (for the analysis) was used and heated in air to 450 °C within 2 h to evaporate the water. Then, the samples were fixed in a Ni-wire cage and were suspended into the melt for 15 min after preheating. The surface morphology of silicon carbide single crystal and dislocation etch pits were observed with the help of a LEXT OLS4000 3D laser confocal microscope and scanning electron microscope (HITACHI, Tokyo, Japan). The LEXT OLS4000 3D laser confocal microscope was manufactured by Olympus, Inc., Tokyo, Japan. A 405 nm laser was used as the source with a laser power of 6 mW. The vertical and transverse resolutions were 0.01 µm and 0.12 µm, respectively. The visible light mode and laser mode were used to obtain the surface morphology and 3D shape of the etch pits, respectively.

## 3. Results and Discussion

### 3.1. An Abnormal Star-Like Defect

The forward blocking and on-state characteristics for 1.0 cm^2^ GTOs were measured by a Keysight B1505 Semiconductor Parameter Analyzer. Most GTO devices have a breakdown voltage of more than 3 KV. However, a few GTO devices have a breakdown voltage of lower than 3 KV, and even close to zero. The number of the 1.0 cm^2^ SiC GTOs on a 4-inch 4H-SiC wafer is 54, and voltage failure GTO devices are scattered throughout the 4-inch 4H-SiC wafer. Therefore, the effect of the device preparation process, such as ion implantation and annealing, on the electrical properties of the device is excluded. A KLA Tencor Candela CS920 (Candela, Los Angeles, America) was used to identify and locate surface defects (triangle defects, carrot defects, and particles) of 4H-SiC epilayers before 1.0 cm^2^ GTOs preparation. Nevertheless, no correlation can be established between device voltage failure and surface defects. In order to study the reasons behind the low breakdown voltage, the defect exposure and characterization of the failure devices were carried out.

**Figure 2:**
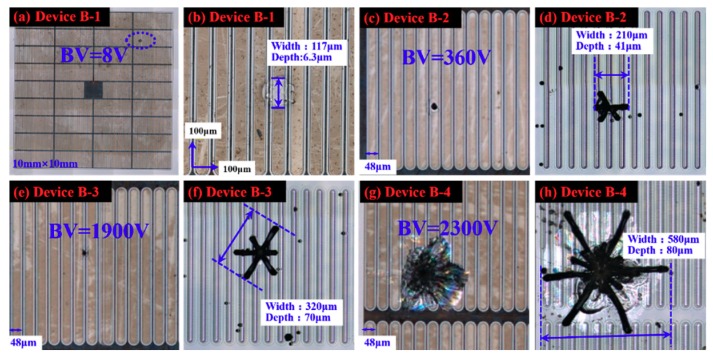
Morphology diagrams after metal removal at room temperature and KOH etching at 450 °C. (**a**,**b**,**c**,**e**,**g**) are morphology diagrams of devices B1–B4 after metal removal; (**d**,**f**,**h**) are morphology diagrams of devices B2–B4 after KOH etching.

Morphology diagrams after metal removal at room temperature and KOH etching at 450 °C for device B1–B4 referral to Table 1 are shown in Figure 2. The Anode/Gate design of 1.0 mm^2^ SiC GTO thyristors fabricated on the same wafer is a fine raster and the width of a single anode is 48 µm, which can be clearly seen in Figure 2b. After metal removal, a large pit on the GTO device exists, which has a breakdown voltage lower than 3 KV. The big pit may be at the anode region, gate region, or across several anode regions from Figure 2b,c,e,g. However, there are no big pits on devices with breakdown voltage higher than 3 KV. For device B-1, the pit on it after metal removal is 117 µm in width and 6.3 µm in depth, as shown in Figure 2b, and it is noteworthy that the big pit runs through three anodes and two gates of a 1.0 cm^2^ SiC GTO. After KOH etching at 450 °C, these big pits observed after metal removal developed into a hexagonal symmetric star-like etch pit with 210–580 µm in width and 41–80 µm in depth, as shown in Figure 2d,f,h. The star-like etch pits run through 4–12 strip anodes of a 1.0 cm^2^ SiC GTO, and one line of a star-like defect is perpendicular to the anode raster. In addition, the angle between the three dislocation lines of the star-like defect is almost 60° and the orientation of the three dislocation lines is consistent with the six-fold symmetry of silicon carbide.

**Figure 3:**
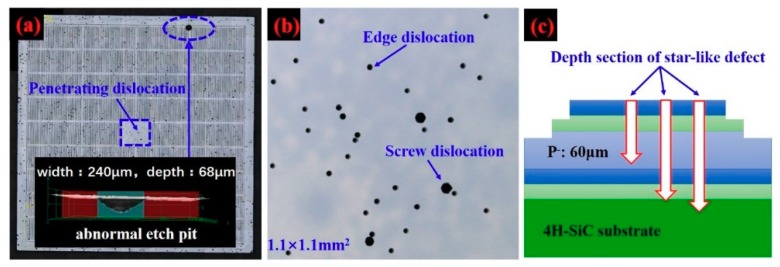
Morphology diagram after KOH etching at 450 °C (**a**). (**b**) a larger view of the central square area on Figure 3a. Schematic cross-section view of star-like defect on a 1.0 cm^2^ SiC GTO (**c**).

Subcircular and hexagonal etching pits with depths of 2 to 7 µm appeared on the Si-face, which correspond to penetrating dislocations in 4H-SiC, as shown in Figure 3b. It has been reported that large hexagonal etch pits without bottoms represent micropipes, middle-sized hexagonal etch pits with bottoms represent screw dislocations, while small hexagonal etch pits with bottoms represent threading edge dislocations [11]. Amazingly, in Figure 3a, the hexagonal symmetric star-like etch pit is much larger than the etch pit of a penetrating dislocation. Furthermore, as for the depth of the star-like etch pits being 41–80 µm and the GTO having a 60 µm drift layer thickness, as shown in Figure 3c and Figure 1, a star-like defect across the drift layer is found and almost extends into the 4H-SiC substrate.

### 3.2. Morphology and Structure of the Star-Like Defect

**Figure 4:**
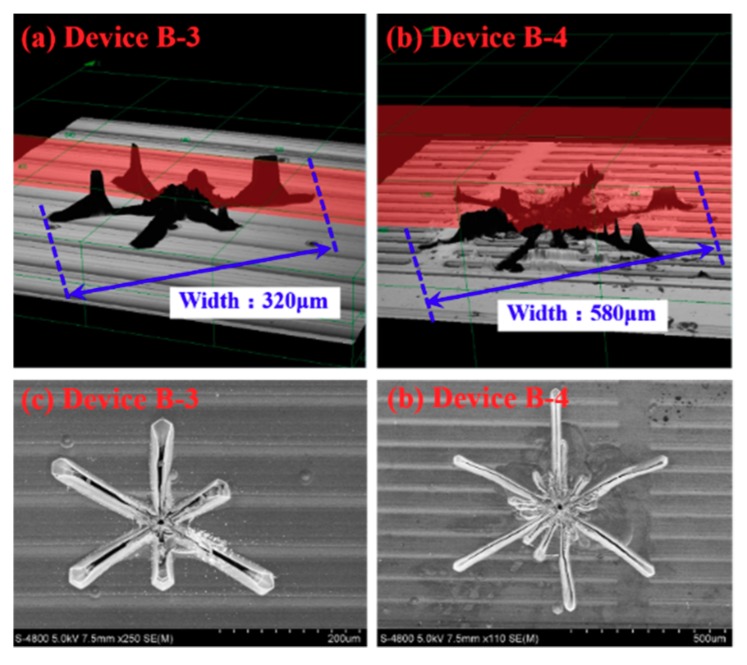
Abnormal etch pit of device B-3 and B-4. (**a**,**b**) are the 3D-shape of the abnormal etch pits measured by a LEXT OLS4000 3D laser confocal microscope, (**c**,**d**) are the topography of the abnormal etch pits measured by Scanning electron microscope (SEM).

Then, the 3D-shape of the abnormal hexagonal symmetric star-like etch pits shown in Figure 2f,h was measured by a LEXT OLS4000 3D laser confocal microscope and scanning electron microscope (SEM), and the results are shown in Figure 4. From Figure 4a,b, it can be found that the hexagonal symmetric star-like defect contains six to seven deep etch pits with a ladder cone shape section. These etch pits are located at the edge as well as the intersections of the three etch lines. According to the typical sectional view of etch pits, the hexagonal etch pits that have a ladder cone shape section correspond to a mixed dislocation with a Burgers vector of $b_{m}=b_{e}\;$*+*$\;b_{s}$, where $b_{e}=\frac{na}{3}\langle 11\bar{2}0\rangle$ and $b_{m}=mc$ (n = 1, 2, 3……; m = 1, 2, 3) [12]. That is to say, the star-like defect consists of several mixed dislocations. In addition, the morphology obtained by SEM is shown in Figure 4c,d, which is consistent with that obtained by the laser confocal microscope. Wu et al. [13,14] reported that they found a new stacking fault in 4H-SiC wafers through synchrotron white-beam x-ray topography. This fault has the shape of a six-pointed star, comprising faults with three different fault vectors of Shockley type. A micropipe is located in the center of the star stacking fault. However, the star-like defect found in our work after KOH etching at 450 °C consists of several mixed dislocations, not stacking faults.

### 3.3. The Influence of the Star-Like Defect on the Forward and Blocking Characteristic of SiC GTOs

**Figure 5:**
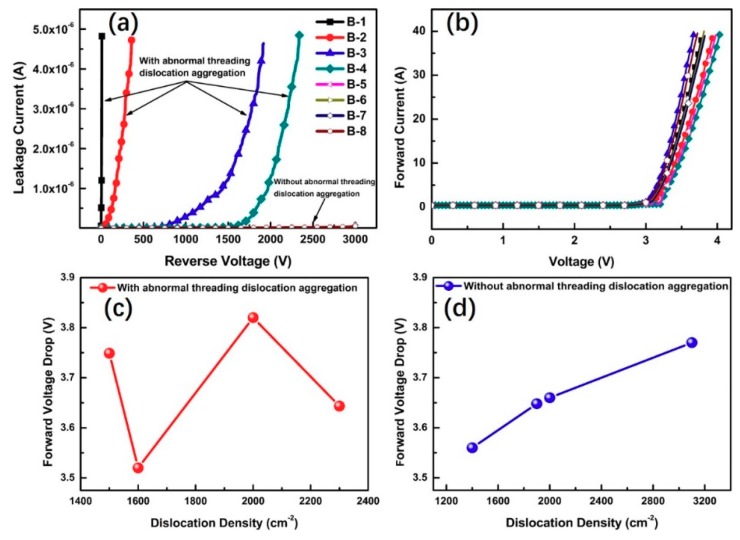
(**a**) reverse I–V characteristics and (**b**) the forward characteristics of anode-gate GTOs for devices B1–B8. The effect of threading dislocation density on forward voltage drop at 100 A/cm^2^ for the GTOs (**c**) with abnormal threading dislocation aggregation, and (**d**) without abnormal threading dislocation aggregation.

Reverse I–V and the forward characteristics of anode-gate GTOs for device B1–B8 were measured, and the result is shown in Figure 5. The leakage current is on the order of 10^−6^ A for devices B1–B4 before voltage failure, while it is about 10^−8^ A for devices B5–B8. The leakage current value of the GTOs with abnormal threading dislocation aggregation is two orders of magnitude higher than the GTOs without abnormal threading dislocation aggregation. In addition, a summary of the breakdown voltage and dislocation density in devices B1–B8 is given in Table 1. Herein, dislocation density was obtained by an average of nine points across the 1.0 mm^2^ GTO [15]. From Table 1, it can be seen that the breakdown voltage shows no essential correlation with the dislocation density. Therefore, it can be inferred that these abnormal star-like etch defects from dislocation aggregation lead to the failure of blocking characteristics, rather than the isolated dislocations. It has been reported that inverted cone-shaped nano-scale pits with a diameter of approximately 200 nm, a depth of approximately 45 nm, were observed at the Schottky junction interface, and it was found that leakage current increases in these diodes due to the concentration of electric fields at the peaks of the pits [16]. This is in a good agreement with the deteriorated blocking characteristics of current GTOs with large abnormal star-like defects.

Figure 5b shows the forward characteristics of anode-gate GTOs with and without abnormal threading dislocation aggregation. There is no essential difference between GTOs with and without abnormal dislocation aggregation. However, the abnormal dislocation aggregation can affect the relationship between voltage drop and dislocation density as shown in Figure 5c,d, which is an interesting phenomenon. For GTOs without abnormal etch pits, a regular increase of forward voltage drop at 100 A/cm^2^ was observed with the increase of threading dislocations density, which can be ascribed to the well-known carrier modulation degradation from enhanced carrier recombination at dislocations, while this correlation disappears in the presence of abnormal etch pits as shown in Figure 4c, which plays a predominant role over dislocations in the blocking characteristics.

In single crystals, a penetrating dislocation occurs due to lattice distortions. As a result, these areas have higher strain energies than perfect single crystal regions. Due to the higher chemical potential, strained areas of single crystals are more liable to chemical attack than non-strained areas. The elastic strain energy of threading screw dislocations (TSDs) is greater than that of threading edge dislocations (TEDs) by a factor of 3 [17]. The exposure and growth of the star-like defect after KOH etching means that the strain energy around the star-like defect is higher than that of the perfect single crystal regions. In addition, the higher stress and strain may be introduced by blocking characteristics measurements. From the above discussion and based on our current understanding of penetrating dislocations in 4H-SiC, we infer that the close penetrating dislocations will aggregate and merge under stress concentration, leading to forming star-like defects and releasing partial stress. Since stress concentration leads to the formation of the abnormal star-like defect after KOH etching, we deduce that the electrical stress also gives rise to the voltage concentration at the defect in the same way, which induces the close penetrating dislocations merge to form star-like defects, and in turn to reduce the breakdown voltage. Similar results have been reported that the breakdown voltages of the diodes with threading screw dislocations and threading edge dislocations were reduced by 15.5% and 6.5%, compared with those of a diode without defects [18].

Based on the above discussion, forward voltage drop induced by an abnormal threading dislocation aggregation in 4H-SiC GTO devices is elucidated. Improving the quality of single crystals and reducing the connection of penetrating dislocations under mechanical or electrical stress are the keys to improving the reliability of SiC GTOs.

## 4. Conclusions

An abnormal star-like defect was found after both metal removal and KOH etching at 450 °C on the failed SiC GTO devices with lower breakdown voltage. It consists of several penetrating dislocations and its orientation is consistent with the six-fold symmetry of silicon carbide. The forward blocking characteristic of 1.0 cm^2^ GTOs is severely deteriorated by this abnormal star-like defect rather than the dislocation in much higher density. As for the forward characteristics, it is found that the influence of dislocation density on the forward voltage drop is affected by the presence of star-like defects from threading dislocation aggregation. The forward voltage drop at 100 A/cm^2^ for GTOs without abnormal etch pits shows a regular increase with the increasing of dislocation density, while this correlation disappears in the presence of abnormal etch pits. In a word, this abnormal star-like defect is a killing defect for both forward and blocking characteristics of SiC GTOs.

## Figures and Tables

**Table 1:** The breakdown voltage blocking voltage (BV), threading dislocation density, abnormal etch pit for devices B1–B8.

Device Number	BV (V)	Dislocation Density (cm^−2^)	Abnormal Etch Pit
Device B-1	8	2300	a star-like etch pit
Device B-2	360	1500	a star-like etch pit
Device B-3	1900	1600	a star-like etch pit
Device B-4	2300	2000	a star-like etch pit
Device B-5	>3000	3100	no
Device B-6	>3000	1900	no
Device B-7	>3000	2000	no
Device B-8	>3000	1400	no
